# Identifying novel risk factors for aneurysmal subarachnoid haemorrhage using machine learning

**DOI:** 10.1038/s41598-025-88826-3

**Published:** 2025-03-18

**Authors:** Jos P. Kanning, Junfeng Wang, Shahab Abtahi, Mirjam I. Geerlings, Ynte M. Ruigrok

**Affiliations:** 1 Department of Neurology and Neurosurgery, UMC Utrecht Brain Center, University Medical Center Utrecht, Utrecht, The Netherlands; 2 Julius Center for Health Sciences and Primary Care, University Medical Center Utrecht, Utrecht University, Utrecht, The Netherlands; 3 Division of Pharmacoepidemiology and Clinical Pharmacology, Utrecht Institute for Pharmaceutical Sciences, Utrecht University, Utrecht, The Netherlands; 4 Department of General Practice, Amsterdam UMC, location University of Amsterdam, Meibergdreef 9, Amsterdam, The Netherlands; 5 Amsterdam Public Health, Aging & Later life, and Personalized Medicine, Amsterdam, The Netherlands; 6 Amsterdam Neuroscience Neurodegeneration, and Mood, Anxiety, Psychosis, Stress, and Sleep, Amsterdam, The Netherlands

**Keywords:** Stroke, Risk factors, Statistics

## Abstract

Aneurysmal subarachnoid haemorrhage (aSAH) is a type of stroke with high mortality and morbidity. This study aimed to identify novel aSAH risk factors by combining machine learning (ML) and traditional statistical methods. Using the UK Biobank, we identified aSAH cases via hospital-based ICD codes and analysed 618 baseline variables covering demographics, lifestyle, medical history, and physical measurements. The CatBoost ML algorithm and Shapley Additive Explanations (SHAP) identified the top 25 variables most influential in predicting aSAH. Logistic regression further described these variables while adjusting for established aSAH risk factors. Among 501,847 participants, 893 aSAH cases were identified. ML identified 214 variables with non-zero SHAP values. Logistic regression of the top 25 variables revealed four potential novel aSAH risk factors. Increased aSAH risk was associated with mean sphered cell volume (OR 1.02, 95% CI 1.00-1.03) and tea intake (OR 1.03, 95% CI 1.01–1.05). Decreased aSAH risk was associated with peak expiratory flow (OR 0.80, 95% CI 0.66–0.96), and haematocrit percentage (OR 0.97, 95% CI 0.95-1.00). Future research should validate these findings and explore the potential non-linear relationships and interactions indicated by the ML models.

**Supplementary Information:**

The online version contains supplementary material available at 10.1038/s41598-025-88826-3.

## Introduction

Aneurysmal subarachnoid haemorrhage (aSAH) is a type of stroke that occurs when an intracranial aneurysm ruptures^1^. Despite accounting for only 10% of all strokes^2^, aSAH is particularly devastating due to its early age of onset and high mortality rate, leading to a number of potential life years lost comparable to those lost to ischaemic stroke, the most common type of stroke^3^. Current understanding of the pathogenesis of aSAH remains limited^4^. Established risk factors include age, female sex, hypertension, smoking, and excessive alcohol consumption^5^. However, prediction models incorporating these established risk factors are only moderately able to discriminate between aSAH cases and controls^6,7^. Thus, knowledge of additional risk factors is required to identify people at risk of aSAH.

Machine learning has emerged as a promising strategy for identifying novel risk factors for various medical conditions^8–10^. Unlike traditional statistical methods, machine learning can process large and diverse datasets, uncover complex non-linear associations and their interactions, and does not require precise model specification before analysis^11–13^. However, one limitation of machine learning is that the models it generates can be difficult to interpret and explain in human terms^14^. Integrating machine learning with traditional statistical methods could provide a comprehensive solution by leveraging the depth and complexity of machine learning analysis while preserving the interpretability of traditional models^8^.

In this study, we aimed to identify new risk factors for aSAH by combining machine learning and statistical methods, using data from the United Kingdom (UK) Biobank’s population-based cohort.

## Results

We identified 893 aSAH patients among 501,847 participants (Table 1). The aSAH patients tended to be older, predominantly female, more frequently current smokers, and more likely to have hypertension compared to the individuals who did not develop aSAH. Finally, aSAH patients were less likely to drink alcohol, but if they did, they drank more often.

**Table 1:** Baseline characteristics.

Variable	No aSAH	aSAH	*p*-value
n, (%)	500,954 (99.82)	893 (0.18)	NA
Age at baseline, mean (SD)	56.53 (8.1)	58.44 (7.64)	< 0.001
Female, n (%)	272,407 (54.49)	567 (63.50)	< 0.001
Hypertension, n (%)	261,542 (52.22)	520 (58.24)	< 0.001
Smoking status, n (%)			< 0.001
Current	52,689 (10.52)	165 (18.48)	
Previous	172,481 (34.43)	298 (33.37)	
Never	272,845 (54.47)	423 (47.37)	
Unknown	2,939 (0.59)	7 (0.78)	
Alcohol use, n (%)			0.001
Daily	101,449 (20.25)	192 (21.50)	
Often	244,069 (48.72)	388 (43.45)	
Rarely	113,475 (22.65)	210 (23.52)	
Never	40,463 (8.08)	101 (11.31)	
Unknown	1,498 (0.30)	2 (0.22)	

P-values are derived from a two-sample t-test for age and Chi-squared tests for the other variables. aSAH = Aneurysmal subarachnoid haemorrhage, OR = Odds-ratio, CI = Confidence interval, SD = Standard deviation.

### Machine learning model

**Fig. 1:**
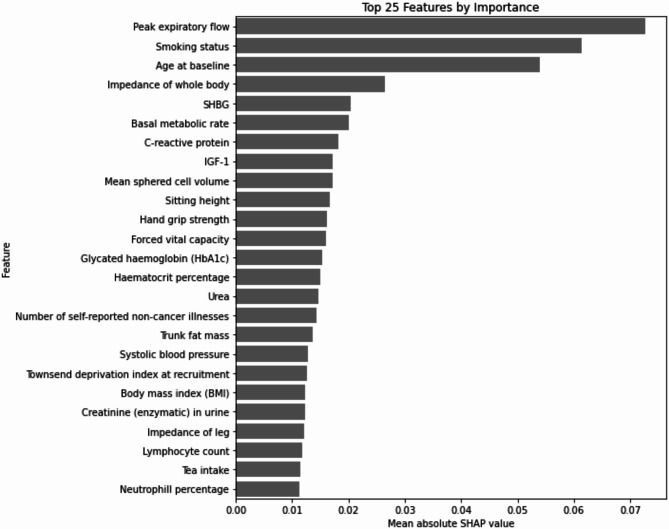
The 25 most important potential risk factors identified by the CatBoost algorithm. SHAP = SHapley Additive exPlanations, SHBG = Sex hormone binding globulin, IGF-1 = Insulin-like growth factor 1.

Initial pre-processing resulted in 235 variables to be used by the CatBoost model of which 214 variables were identified with a mean absolute SHAP value greater than 0 (Supplementary Table 1). The 25 variables with the highest mean absolute SHAP values were: peak expiratory flow, smoking status, age at baseline, impedance of whole body, sex hormone binding globulin (SHBG), basal metabolic rate, C-reactive protein (CRP), insulin-like growth factor 1 (IGF-1), mean sphered cell volume, sitting height, hand grip strength, forced vital capacity, glycated haemoglobin (HbA1c), haematocrit percentage, urea, number of self-reported non-cancer illnesses, trunk fat mass, systolic blood pressure, Townsend deprivation index at recruitment, body mass index (BMI), creatinine (enzymatic) in urine, impedance of leg, lymphocyte count, tea intake, and neutrophil percentage (Fig. 1).

## Traditional statistical model

We did not further investigate age at baseline and smoking status, as they were already included as established risk factors. We also excluded whole body impedance, basal metabolic rate, and trunk fat mass from our study due to their high variance inflation factors, leaving 20 potential risk factors for further analysis (Table 2). A brief description of these potential risk factors can be found in Supplementary Table 2.

**Table 2:** The potential aneurysmal subarachnoid haemorrhage (aSAH) risk factors analysed by traditional statistical methods.

Potential risk factors	No aSAH	aSAH	Unadjusted OR (95% CI)	Adjusted* OR (95% CI)
Log(Peak expiratory flow), mean (SD)	5.87 (0.40)	5.77 (0.45)	0.60** (0.53–0.69)	0.80** (0.66–0.96)
Log(SHBG), mean (SD)	3.81 (0.51)	3.91 (0.51)	1.46** (1.26–1.69)	1.29** (1.09–1.53)
Log(C-reactive protein), mean (SD)	0.31 (1.04)	0.42 (1.03)	1.09** (1.02–1.17)	1.01 (0.94–1.08)
IGF-1, mean (SD)	21.37 (5.58)	20.70 (5.64)	0.98** (0.97–0.99)	1.00 (0.98–1.01)
Mean sphered cell volume, mean (SD)	82.79 (5.14)	83.52 (5.17)	1.03** (1.01–1.04)	1.02** (1.00–1.03)
Sitting height, mean (SD)	89.15 (4.88)	88.26 (4.86)	0.96** (0.95–0.97)	0.99 (0.97–1.01)
Hand grip strength, mean (SD)	30.61 (11.03)	28.60 (11.17)	0.98** (0.98–0.99)	1.00 (0.99–1.01)
Log(Forced vital capacity), mean (SD)	1.26 (0.29)	1.19 (0.31)	0.51** (0.42–0.62)	0.92 (0.68–1.25)
Glycated haemoglobin (HbA1c), mean (SD)	35.49 (4.50)	35.74 (4.30)	1.01 (1.00–1.02)	0.99 (0.97–1.00)
Haematocrit percentage, mean (SD)	41.08 (3.54)	40.63 (3.35)	0.96** (0.95–0.98)	0.97** (0.95–1.00)
Urea, mean (SD)	5.37 (1.28)	5.47 (1.37)	1.06** (1.00–1.11)	1.04 (0.99–1.10)
Number of self-reported non-cancer illnesses, mean (SD)	1.86 (1.87)	2.16 (2.26)	1.08** (1.05–1.11)	1.03 (1.00–1.07)
Systolic blood pressure, mean (SD)	137.81 (18.68)	140.25 (18.62)	1.01** (1.00–1.01)	1.00 (1.00–1.01)
Townsend deprivation index at recruitment, mean (SD)	−1.29 (3.09)	−1.15 (3.16)	1.01 (0.99–1.04)	1.00 (0.98–1.02)
Body mass index (BMI), mean (SD)	27.43 (4.80)	26.94 (4.79)	0.98** (0.96–0.99)	0.97** (0.96–0.99)
Log(Creatinine (enzymatic) in urine), mean (SD)	8.88 (0.68)	8.83 (0.68)	0.91 (0.82–1.00)	1.01 (0.91–1.12)
Impedance of leg, mean (SD)	247.36 (35.27)	252.11 (36.28)	1.00** (1.00–1.01)	1.00** (1.00–1.01)
Lymphocyte count, mean (SD)	1.94 (0.60)	1.97 (0.65)	1.08 (0.97–1.21)	0.94 (0.84–1.05)
Tea intake, mean (SD)	3.41 (2.90)	3.72 (3.36)	1.03** (1.01–1.05)	1.03** (1.01–1.05)
Neutrophil percentage, mean (SD)	60.88 (8.52)	60.89 (9.21)	1.00 (0.99–1.01)	1.00 (0.99–1.01)

All odds ratios for numerical variables indicate the increased aSAH risk associated with a 1 standard deviation increase of that variable. *: Adjusted for age at baseline, female sex, hypertension, smoking status, and alcohol use. **: Statistically significant at a threshold of *p* < 0.05 (indicated in bold). OR = Odds-ratio, CI = Confidence interval, SD = Standard deviation, SHBG = Sex hormone binding globulin, IGF-1 = Insulin-like growth factor 1.

We identified 8 of the 20 variables that were univariably associated with an increased risk of aSAH. These included log-transformed SHBG levels (OR 1.46, 95% CI 1.26–1.69), log-transformed CRP levels (OR 1.09, 95% CI 1.02–1.17), mean sphered cell volume (OR 1.03, 95% CI 1.01–1.04), urea (OR 1.06, 95% CI 1.00–1.11), number of self-reported non-cancer illnesses (OR 1.08, 95% CI 1.05–1.11), systolic blood pressure (OR 1.01, 95% CI 1.00–1.01), impedance of leg (OR 1.00, 95% CI 1.00–1.01), and tea intake (OR 1.03, 95% CI 1.01–1.05). After adjusting for established risk factors, the associations remained statistically significant for log-transformed SHBG levels (OR 1.29, 95% CI 1.09–1.53), mean sphered cell volume (OR 1.02, 95% CI 1.00–1.03), impedance of leg (OR 1.00, 95% CI 1.00–1.01), and tea intake (OR 1.03, 95% CI 1.01–1.05). A sensitivity analysis revealed that compared to low tea intake (less than 2 cups a day), medium tea intake (between 2 and 5 cups) was associated with a decreased aSAH risk (OR 0.89, 95% CI 0.76–1.05), whereas high intake was associated with an increased risk (OR 1.11, 95% CI 0.94–1.32). We found that the increased aSAH risk associated with high tea intake was more pronounced in women than in men (Supplementary Fig. 2).

We also found 7 of the 20 variables to be univariably associated with a decreased aSAH risk: log-transformed peak expiratory flow (OR 0.60, 95% CI 0.53–0.69), IGF-1 (OR 0.98, 95% CI 0.97–0.99), sitting height (OR 0.96, 95% CI 0.95–0.97), hand grip strength (OR 0.98, 95% CI 0.98–0.99), log-transformed forced vital capacity (OR 0.51, 95% CI 0.42–0.62), haematocrit percentage (OR 0.96, 95% CI 0.95–0.98), and BMI (OR 0.98, 95% CI 0.96–0.99). After adjusting for established risk factors, the associations remained statistically significant for log-transformed peak expiratory flow (OR 0.80, 95% CI 0.66–0.96), haematocrit percentage (OR 0.97, 95% CI 0.95–1.00), and BMI (OR 0.97, 95% CI 0.96–0.99). Finally, we found an interaction between haematocrit percentage and age, with older individuals with low haematocrit percentage at the highest risk of aSAH (Supplementary Fig. 3).

For the remaining 5 variables—HbA1c, Townsend deprivation index, log-transformed creatinine in urine, lymphocyte count, and neutrophil percentage—we did not find any statistically significant associations with aSAH.

## Discussion

Using a combination of machine learning and traditional statistical approaches, we identified four variables associated with an increased risk of aSAH. These included log-transformed SHBG, mean sphered cell volume, leg impedance, and tea consumption. In contrast, three variables were linked to a decreased risk of aSAH: log-transformed peak expiratory flow, haematocrit percentage, and BMI.

Our analysis identified two new potential risk factors for aSAH. The first, mean sphered cell volume, measures red blood cells in a spherical state. Although no prior studies have linked mean cell sphered volume to aSAH, it could be associated with aSAH via macrocytosis, which leads to increased blood viscosity and possibly a higher risk of rupture^15^. However, the small effect size we observed for this risk factor may suggest alternative mechanisms. For example, the effect may be mediated through other risk factors associated with high mean cell sphered volume, such as vitamin B12 deficiency^16^. In turn, vitamin B12 deficiency may be associated with extreme forms of the established aSAH risk factors of alcohol abuse and smoking^17^. Although we have adjusted for alcohol use and smoking in our analysis, there remains a risk of residual confounding of their extreme forms. Finally, our research suggests an increased risk of aSAH with high tea consumption, contrasting with studies indicating no association or a reduced risk^18–20^. This discrepancy might be due to differences in the amount of tea consumed. For instance, tea intake in the UK Biobank averages 3.5 cups a day, considerably higher than the at least 1 cup a day defined in other studies. Our sensitivity analysis confirmed this, with a reduced aSAH risk observed for moderate tea intake, and an increased aSAH risk for high intake. Moderate tea consumption may reduce aSAH risk due to antioxidants and anti-inflammatory compounds in tea that improve vascular health and lower blood pressure^18^. These compounds may strengthen blood vessel walls, reducing the likelihood of aneurysms. However, excessive tea intake could increase aSAH risk because high levels of caffeine can elevate blood pressure and induce vascular stress^21^.

Our analysis also identified two new variables potentially associated with a decreased aSAH risk. We found an inverse relationship between peak expiratory flow and the risk of aSAH. Peak expiratory flow is an indicator of lung function which measures the fastest speed at which a person can exhale air after a maximal inhalation. Similar inverse associations between stroke risk and peak expiratory flow have been documented in previous studies^22,23^. Although peak expiratory flow is commonly linked with cardiovascular risk factors like hypertension and smoking^24^, our findings indicate an association even after adjusting for these factors. One possible explanation is that low peak expiratory flow may reflect diminished lung function and possibly chronic hypoxia, which can contribute to vascular remodelling and alterations in blood pressure regulation^25^. This condition could lead to changes in brain blood vessels, increasing their susceptibility to aneurysm formation or exacerbating existing arterial weaknesses. Finally, we identified an inverse relationship between the risk of aSAH and haematocrit percentage, which is the proportion of red blood cells in the bloodstream. Despite previous research reporting no link between aSAH occurrence and haematocrit levels^26^, these levels are often low in patients at hospital admission and can indicate a higher risk of death^27^.

Our results have similarly highlighted several potential risk factors for aSAH that were previously suggested by research but not conclusively established. These include BMI and leg impedance, b relating to body size. The data on the relationship between body size and aSAH risk is inconsistent^5,28,29^, similar to our study. We found a decreased aSAH risk associated with BMI and a small increased risk associated with leg impedance. It has been speculated that very lean individuals might have nutritional deficiencies predisposing them to aSAH^26^. Alternatively, the association between aSAH and body size might reflect epidemiological biases such as unmeasured confounding or selection bias^30^.

Our analysis also corroborates prior research. The CatBoost machine learning algorithm identified age and smoking status as important predictors, a finding supported by our statistical analysis. Although the machine learning model did not directly identify female sex, a well-established risk factor^5^, it did identify SHBG as important. SHBG is a liver-produced protein that binds to sex hormones such as testosterone and oestrogen, regulating their availability in the body. There is evidence that the elevated aSAH risk in women may be hormonally driven^31^, making SHBG levels, or other hormone-related variables, potentially more relevant predictors of aSAH risk than merely biological sex. Similarly, the Catboost model favoured numerical variables such as systolic blood pressure over binary ones like the presence or absence of hypertension. Contrary to initial expectations, only a slight increase in aSAH risk was observed among heavy drinkers, which lost statistical significance after adjusting for other risk factors. The association between alcohol use and aSAH is still under investigation^28^, and there is some evidence that the association only exists for excessive use^32^. Our categorical definition of alcohol use based on frequency of use may have missed important information on current or past amounts of alcohol intake. Intriguingly, those who reported never drinking alcohol showed the highest aSAH risk, which could reflect prior excessive consumption or other health issues leading them to abstain.

The CatBoost machine learning algorithm identified several variables that did not show statistically significant associations in the traditional statistical model. These included CRP, forced vital capacity, HbA1c, Townsend deprivation index, creatinine, lymphocyte count, and neutrophil percentage. These variables might exhibit non-linear associations with aSAH or depend on interaction terms, making them detectable by CatBoost but not by logistic regression. For example, HbA1c may only relate to aSAH beyond a certain threshold, as aSAH is associated with diabetes^5^. Similarly, variables such as forced vital capacity, hand grip strength, urea levels, and IGF-1, which were univariably associated with aSAH but not after adjustment for established risk factors, may be either confounded or indicative of an unknown mediator role. For example, the observed increased aSAH risk in smokers might be partially explained by elevated CRP levels^33^. These are observational findings, however, and further validation is necessary to substantiate these claims.

This study has several strengths. Firstly, the use of a large dataset, including numerous individuals and variables, facilitated the identification of sufficient cases and variables for a hypothesis-generating approach. Additionally, variables in the UK Biobank are systematically assessed for each individual at baseline, enabling us to evaluate the prognostic significance of each variable. This systematic assessment of variables also allowed for proper adjustment of established risk factors, thereby reducing false positives. Finally, by integrating machine learning with statistical methods, we were able to both filter and characterise the variables associated with aSAH. Relying solely on machine learning would not have permitted quantification of predictor effects, just as relying solely on statistical methods would have precluded analysis of the entire dataset.

This study also had several limitations. First, the rarity of aSAH resulted in poor model performance in the traditional statistical models. Consequently, automatic model selection procedures often yielded an empty null model, restricting our statistical analysis. This limitation confined our statistical modelling to linear associations, as exploring non-linear relationships or interactions was not feasible. Efforts to address class imbalance through sampling or weighting methods produced unreliable SHAP values, failing to identify established aSAH risk factors such as age and smoking. As a result, we chose to present our findings without adjustments for class imbalance. Moreover, the rarity of the condition necessitated analysing the entire dataset rather than dividing it into development and validation cohorts, as is typical in machine learning studies^34^. This limitation meant we could conduct only minimal internal validation, and our results might not be generalisable to an external dataset. Another limitation is the demographic composition of the UK Biobank participants, who are predominantly older, Caucasian, and from upper-middle-class backgrounds^35^. This demographic skew may limit the generalizability of our findings to other populations. We similarly faced limitations due to a lack of radiographic information, preventing us from verifying whether individuals with aSAH-related ICD codes actually had a ruptured aneurysm. Consequently, we were also unable to investigate known aneurysmal risk factors such as aneurysm size and shape. Finally, we did not adjust for multiple comparisons, as the goal of our study was to generate hypotheses by identifying as many potential risk factors for aSAH as possible, without assuming causal relationships among these variables. Furthermore, the selection of potential risk factors was not based on statistical significance. Nevertheless, the lack of such adjustments stress the need to externally validate these initial signals to ensure their reliability and generalizability.

In conclusion, we have identified four new potential risk factors for aSAH. Mean sphered cell volume and tea intake were associated with increased aSAH risk. In contrast, peak expiratory flow and haematocrit percentage were associated with decreased aSAH risk. Our research builds upon previous studies by providing further evidence that body size is associated with aSAH risk, by suggesting that hormonal levels may partially explain the higher aSAH risk among women, and by confirming previously established risk factors. Future studies should use larger sample sizes to validate these preliminary findings. Additionally, special attention should be given to factors identified by the Catboost algorithm that were not statistically significant in the logistic regression model. This could indicate potential non-linearities or interaction effects of these factors which are detectable by CatBoost but not by logistic regression.

## Methods

### Data source

The UK Biobank, an ongoing large-scale prospective population-based cohort study, has collected health-related data from over 500,000 participants, recruited between 2006 and 2010, who were aged 37 to 73 years at baseline^36^. A systematic medical history was taken for each participant on their assessment date, including touchscreen questionnaires, verbal interviews, physical measurements, and biological sample assays. Additionally, the UK Biobank performs linkage to external hospital inpatient records, which include details on admission dates, diagnoses (including underlying conditions), procedures, and discharge information.

### Outcome

We defined all aSAH cases between January 1, 1997, and October 31, 2022, using the hospital-based International Classification of Diseases, 9th Revision (ICD-9) code ‘430’ and 10th Revision (ICD-10) codes I60.0-I60.9. We specifically included codes I60.8 (“Other nontraumatic subarachnoid haemorrhage”) and I60.9 (“Nontraumatic subarachnoid haemorrhage, unspecified”) in our definition of aSAH because there is a high likelihood that these codes also include aSAH cases within the UK Biobank data. Typically, non-aneurysmal subarachnoid haemorrhages account for about 10–15% of all subarachnoid haemorrhage cases^37^. However, in the UK Biobank, codes I60.8 and I60.9 represent 59.4% of all subarachnoid haemorrhage cases, with 530 out of 893 cases, suggesting a probable inclusion of aSAH cases under these codes. Individuals diagnosed with aSAH before their assessment date were excluded from further analysis.

### Predictors

We included all variables that were systematically assessed on the baseline assessment visit and available for at least 80% of the total cohort. This selection encompassed 618 variables, spanning categories such as patient characteristics (e.g., age, sex), sociodemographic factors (e.g., education, ethnicity), lifestyle factors (e.g., smoking status, diet), family and medical history, medication use, physical measurements (e.g., weight, blood pressure), blood assays, and environmental factors (e.g., noise and air pollution of residence area).

In the pre-processing of data, variables that allowed multiple responses from participants were converted into binary vectors, with each potential response encoded as a separate variable. For example, in response to the original question, “Vascular/heart problems diagnosed by a doctor,” participants could select from four options: heart attack, angina, stroke, and high blood pressure. Consequently, we created four distinct binary (yes/no) variables. For variables involving multiple same-day measures, such as systolic blood pressure, the values were averaged to create a single variable. In cases of highly correlated variables (absolute Pearson correlation greater than 0.90), we retained the variable with the fewest missing data points. We considered answers such as “Do not know” and “Prefer not to answer” as missing values. We refrained from removing outliers and instead relied on the outlier checks already implemented by the UK Biobank^38^. For instance, responses to “How many cups of tea do you drink each day?” indicating more than 99 per day were automatically excluded, while responses over 20 prompted verification from participants.

Based on the literature, we identified five established risk factors for aSAH: age at baseline, female sex, hypertension, smoking, and alcohol use^5^. Hypertension was defined as meeting one or more of the following criteria: an average systolic blood pressure over 140, diastolic blood pressure over 90, taking blood pressure medication, or having a doctor-diagnosed condition. Smoking status was categorised as ‘never,’ ‘previous,’ or ‘current.’ Alcohol consumption was categorised as ‘never,’ ‘rarely’ (defined as one to three times a month, or on special occasions), ‘often’ (defined as one to four times a week), and ‘daily.’

### Statistical analysis

**Fig. 2:**
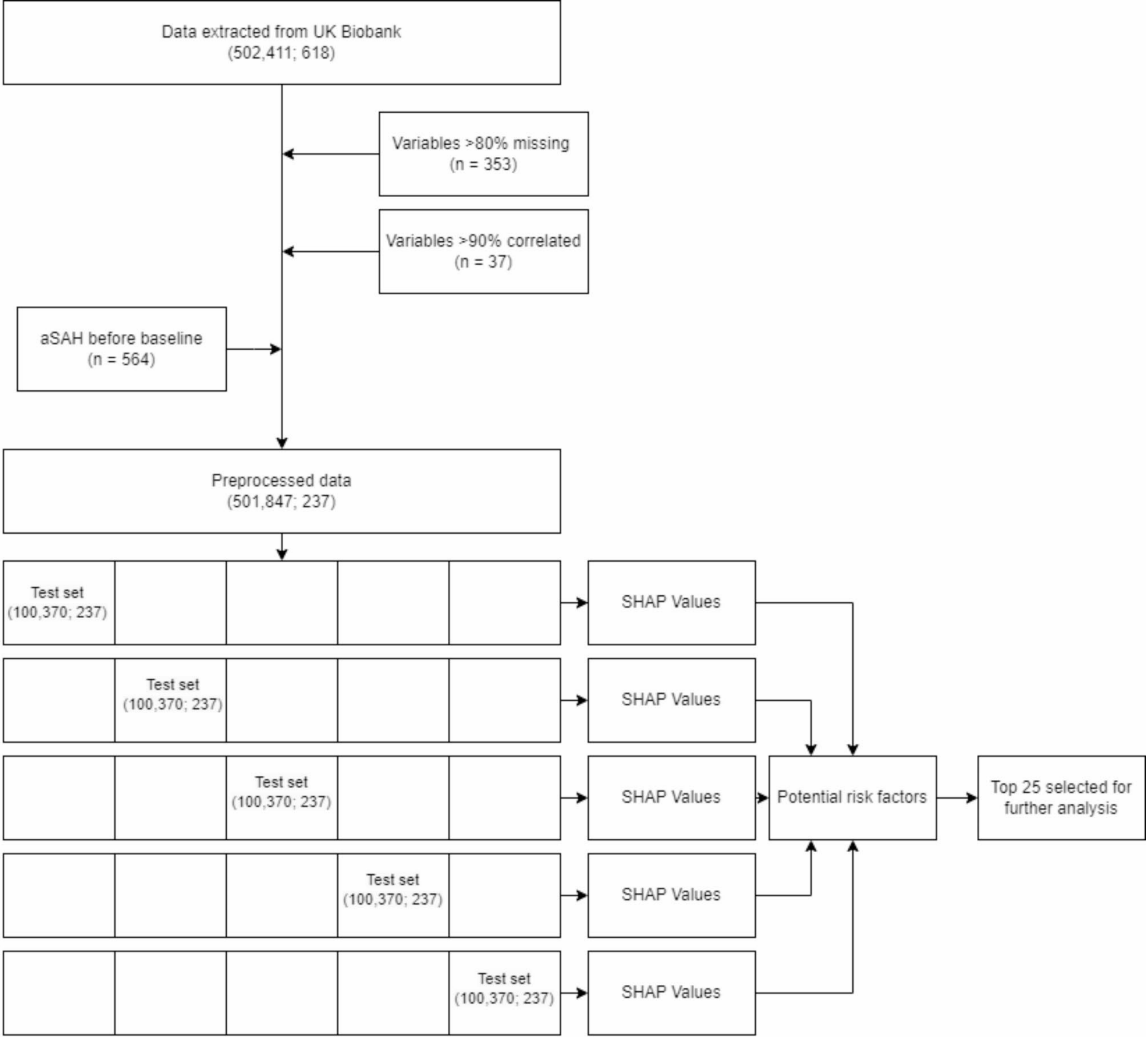
Flowchart of study design. Numbers in parentheses indicate the number of rows (i.e. participants) and columns (i.e. variables) respectively. aSAH = aneurysmal subarachnoid haemorrhage, SHAP = SHapley Additive exPlanations.

Our analysis consisted of two parts (Fig. 2). In the first part, we used a machine learning algorithm to identify potential risk factors for aSAH without predefined hypotheses. In the second part, we used logistic regression to examine and quantify these potential risk factors, while adjusting for established risk factors.

### Machine learning algorithm

In the first part, we used the CatBoost machine learning algorithm to identify potential aSAH risk factors^39^. CatBoost is a gradient-boosting algorithm that operates on decision trees and is designed to process both numerical and categorical data without extensive pre-processing. A key advantage of CatBoost is its capability to automatically handle missing values without the need for imputation. We randomly divided the dataset into five equal-sized folds, each containing a similar proportion of aSAH cases. We trained a CatBoost model on four folds and reserved the fifth for validation. This cross-validation process was repeated until each fold served as the validation fold once, maximising the area under the curve (AUC) score for each validation fold. To assess variable importance, we calculated Shapley Additive Explanations (SHAP) values for each of the five models^40^. We then computed the average of the mean absolute SHAP values across the folds to identify the 25 variables with the highest mean absolute SHAP values. These variables represent the variables that had the greatest influence on the predicted probability of aSAH.

### Traditional statistical model

In the second part, we used traditional statistical methods to further analyse the 25 variables with the highest mean absolute SHAP values, identified in the first part. We did not further analyse established risk factors when identified by the CatBoost model. We additionally removed variables with a variance inflation factor higher than 5 to account for multicollinearity. We addressed missing data by using multiple imputation by chained equations (MICE) with five iterations and five imputed datasets^41^. We then visually examined the distribution of continuous variables and applied a logarithmic transformation to those variables that exhibited severe right-skewness. We proceeded to fit both univariable (unadjusted) logistic regression models and models adjusted for established risk factors to the imputed datasets. In the adjusted models, we designated “never” as the reference category for smoking status and “rarely” for alcohol use. We present differences in summary statistics for the established risk factors, using two-sample t-tests for numerical variables and chi-squared tests for the categorical variables. Finally, we presented the unadjusted and adjusted odds ratios (OR), 95% confidence intervals (95% CI), and p-values for each potential risk factor for aSAH by pooling the datasets and using Rubin’s rules^42^. We report the ORs as the increased aSAH risk associated with a 1 standard deviation increase of that variable to account for different variable scales. We additionally investigated interaction effects between the established aSAH risk factors and potentially novel aSAH risk factors. The machine learning model was developed in Python (version 3.13)^43^, using the packages pandas^44^, numpy^45^, catboost^39^, shap^40^, and scikit-learn^46^. The traditional statistical models were developed in R (version 4.4.0)^47^, using the ggplot2^48^, dplyr^49^, and mice packages^50^.

### Reporting standards

All results are reported according to the Strengthening the Reporting of Observational Studies in Epidemiology (STROBE) guidelines^51^.

The UK Biobank received ethics approval from the North West Multi-Center Research Ethics Committee (REC No. 16/NW/0274), and all participants provided written electronic informed consent.

## Electronic supplementary material

Below is the link to the electronic supplementary material.

Supplementary Material 1

## Data Availability

This research was conducted using the UK Biobank resource under application number 2532. The datasets are not publicly available but can be accessed via the UK Biobank data access process. More details are available at http://www.ukbiobank.ac.uk/register-apply/.
