# Supplementary material for: Identifying novel risk factors for aneurysmal subarachnoid haemorrhage using machine learning
Source: Sci Rep. 2025 Mar 18;15:9256. doi: 10.1038/s41598-025-88826-3 (PMC11920089; doi:10.1038/s41598-025-88826-3)
Supplement: Supplementary file 1 — Supplementary Material 1 [file 41598_2025_88826_MOESM1_ESM.docx]

**Supplementary Information**

**Supplementary Figure 1.** Distributions of potential risk factors.


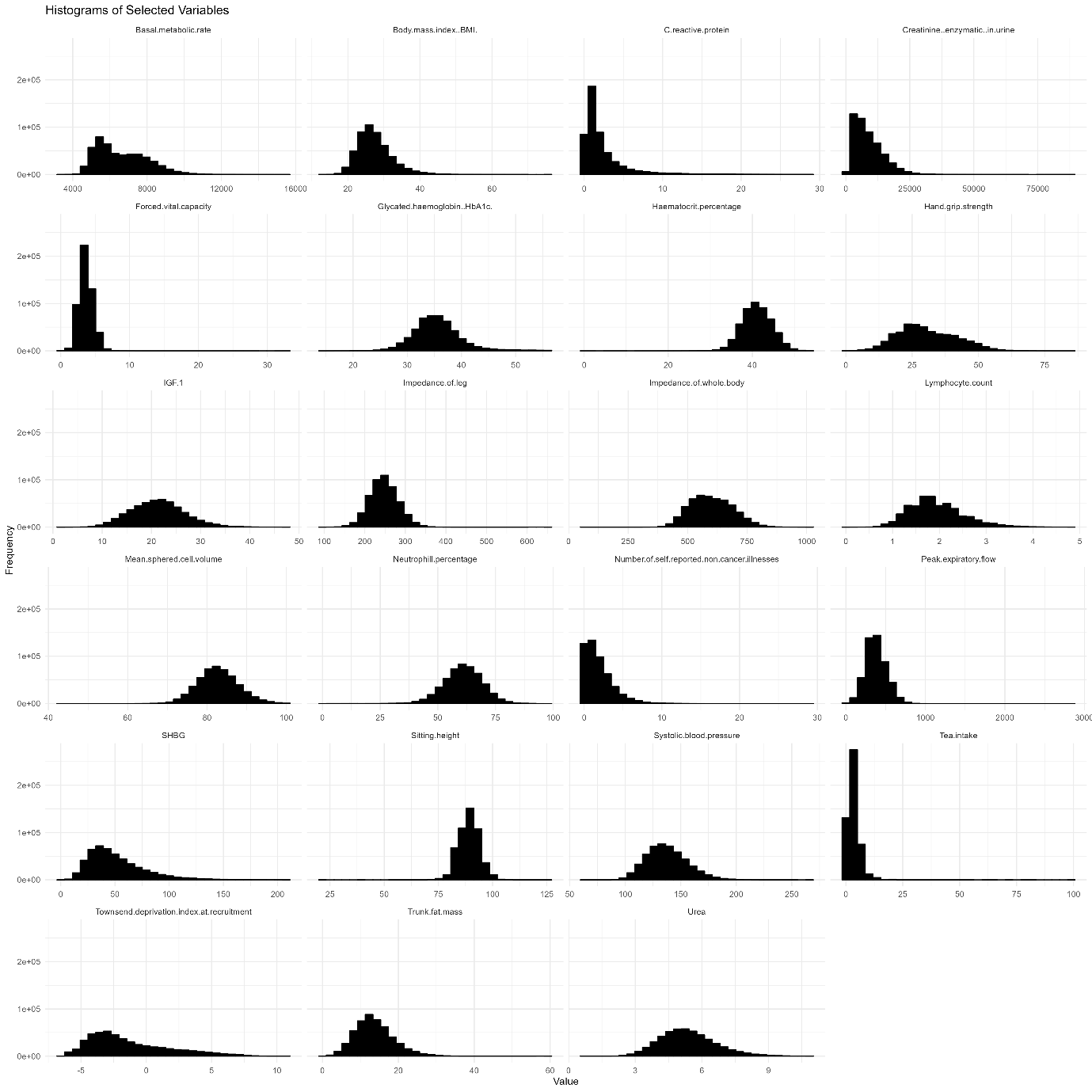


SHBG = Sex hormone binding globulin, IGF-1 = Insulin-like growth factor.

**Supplementary Figure 2.** The interaction between haematocrit percentage and age in relationship to the predicted probability aneurysmal subarachnoid haemorrhage (aSAH).


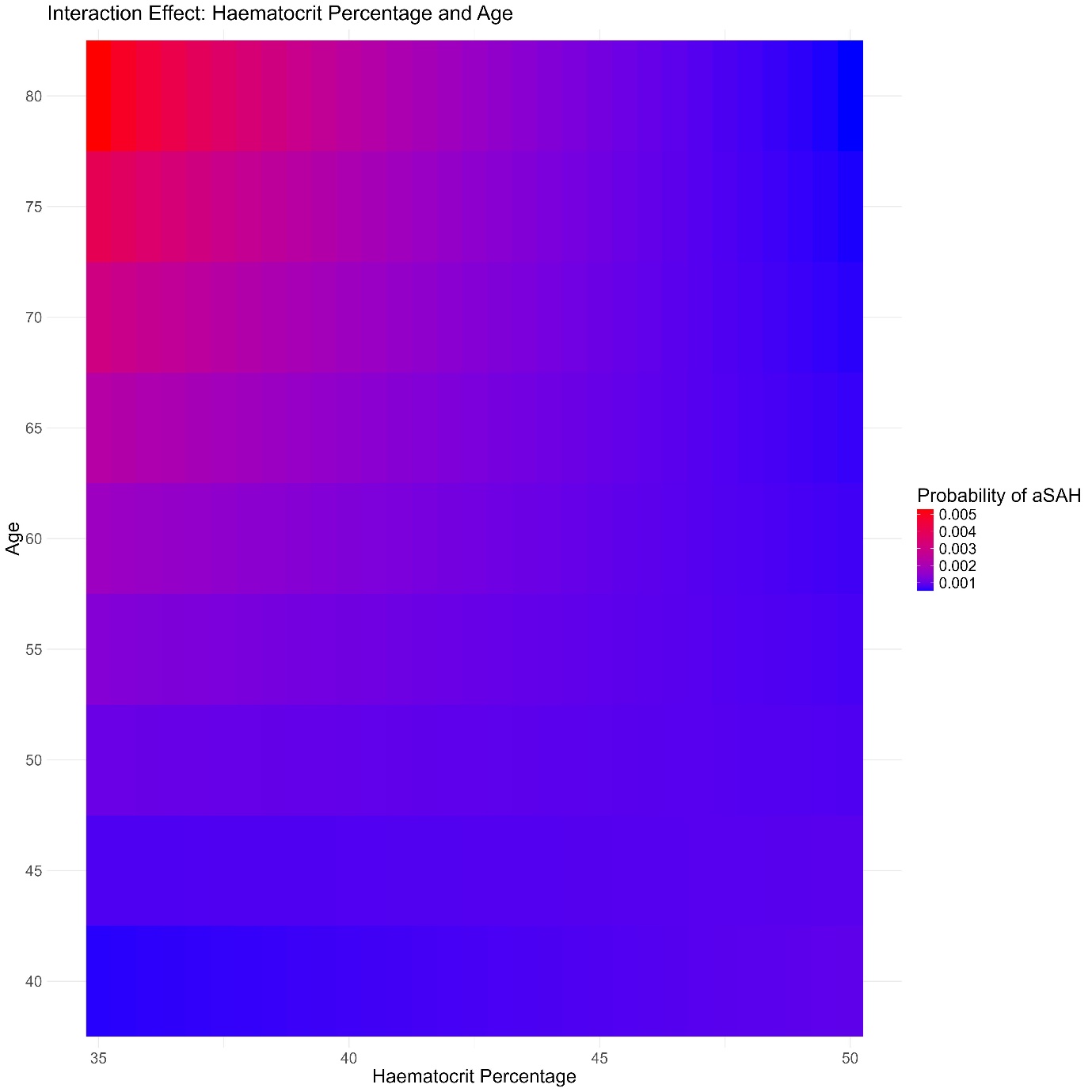


The probabilities of aSAH are derived from a model in which the other established risk factors (i.e. biological sex, hypertension, alcohol use, and smoking) are held constant.

**Supplementary Figure 3**. The interaction between tea intake and biological sex in relationship to the predicted probability aneurysmal subarachnoid haemorrhage (aSAH).


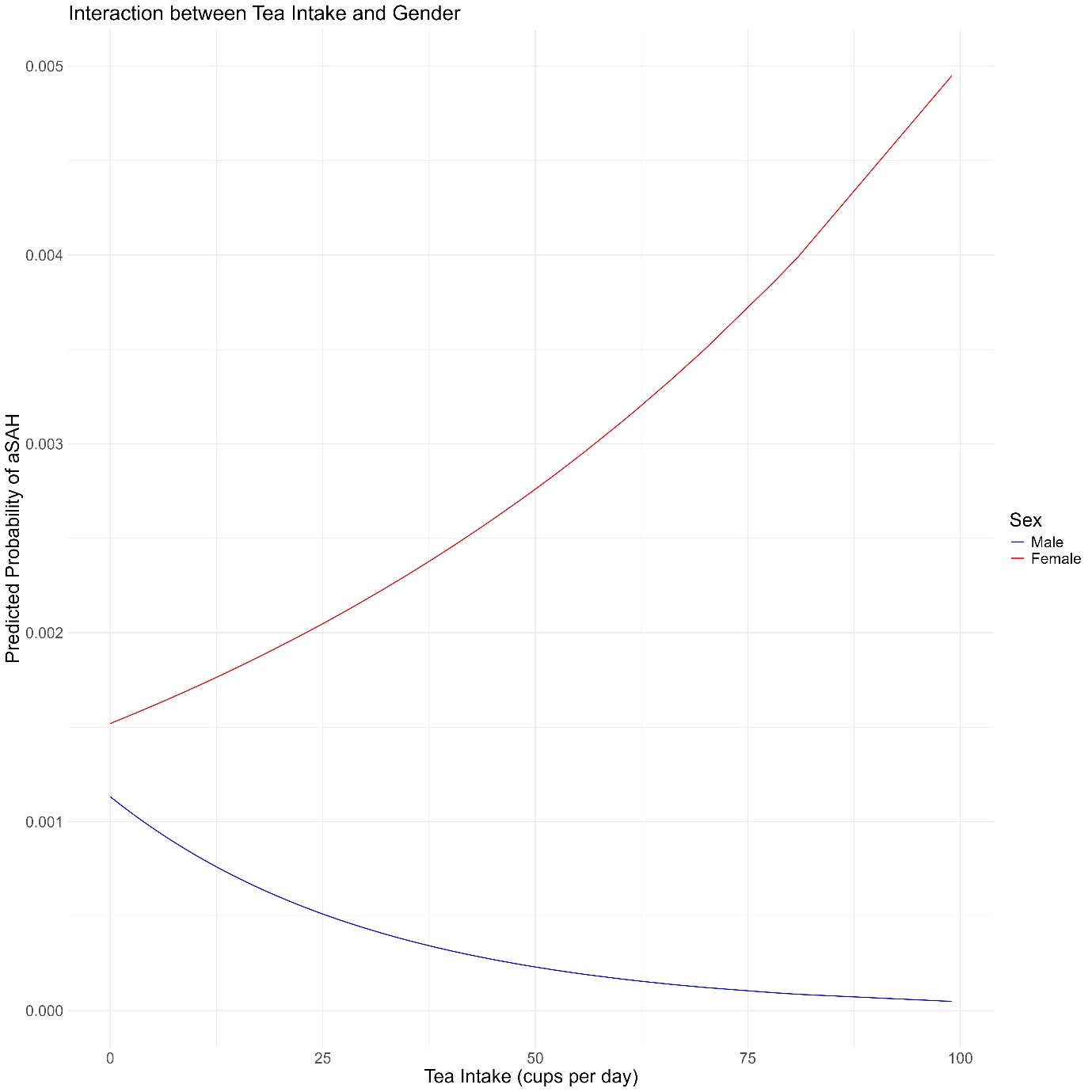


The probabilities of aSAH are derived from a model in which the other established risk factors (i.e. age, hypertension, alcohol use, and smoking) are held constant.

**Supplementary Table 1**. All variables identified by the CatBoost machine learning algorithm with a mean average SHapley Additive Explanations (SHAP) value larger than 0, sorted from most to least important.

| **Variable** | **Mean average SHAP value** |
| --- | --- |
| Peak expiratory flow | 0.073 |
| Smoking status | 0.061 |
| Age at baseline | 0.054 |
| Impedance of whole body | 0.026 |
| SHBG | 0.020 |
| Basal metabolic rate | 0.020 |
| C-reactive protein | 0.018 |
| IGF-1 | 0.017 |
| Mean sphered cell volume | 0.017 |
| Sitting height | 0.017 |
| Hand grip strength | 0.016 |
| Forced vital capacity | 0.016 |
| Glycated haemoglobin (HbA1c) | 0.015 |
| Haematocrit percentage | 0.015 |
| Urea | 0.015 |
| Number of self-reported non-cancer illnesses | 0.014 |
| Trunk fat mass | 0.014 |
| Systolic blood pressure | 0.013 |
| Townsend deprivation index at recruitment | 0.013 |
| Body mass index (BMI) | 0.012 |
| Creatinine (enzymatic) in urine | 0.012 |
| Impedance of leg | 0.012 |
| Lymphocyte count | 0.012 |
| Tea intake | 0.011 |
| Neutrophill percentage | 0.011 |
| Mean corpuscular volume | 0.011 |
| Number of operations, self-reported | 0.011 |
| Exposure to tobacco smoke at home | 0.011 |
| Total protein | 0.010 |
| High light scatter reticulocyte percentage | 0.010 |
| Alanine aminotransferase | 0.010 |
| Average 24-hour sound level of noise pollution | 0.010 |
| Nitrogen dioxide air pollution; 2005 | 0.010 |
| Number in household | 0.009 |
| Triglycerides | 0.009 |
| Particulate matter air pollution (pm10); 2010 | 0.009 |
| Phosphate | 0.009 |
| Particulate matter air pollution (pm10); 2007 | 0.008 |
| Family history of stroke | 0.008 |
| Vitamin D | 0.008 |
| MET minutes per week for walking | 0.008 |
| Mean time to correctly identify matches | 0.007 |
| Nitrogen oxides air pollution; 2010 | 0.007 |
| Mean corpuscular haemoglobin | 0.007 |
| Bread intake | 0.007 |
| Calcium (in blood) | 0.007 |
| Urate | 0.007 |
| Mean reticulocyte volume | 0.007 |
| Potassium in urine | 0.007 |
| Mean corpuscular haemoglobin concentration | 0.007 |
| Traffic intensity on the nearest major road | 0.007 |
| Seated height | 0.006 |
| Creatinine | 0.006 |
| White blood cell (leukocyte) count | 0.006 |
| Lifetime number of sexual partners | 0.006 |
| Mean platelet (thrombocyte) volume | 0.006 |
| Knee pain | 0.006 |
| Particulate matter air pollution (pm2.5) absorbance; 2010 | 0.006 |
| Diastolic blood pressure | 0.006 |
| Particulate matter air pollution 2.5-10um; 2010 | 0.006 |
| Platelet distribution width | 0.005 |
| MET minutes per week for vigorous activity | 0.005 |
| Eosinophill percentage | 0.005 |
| Gamma glutamyltransferase | 0.005 |
| Neutrophill count | 0.005 |
| Glucose | 0.005 |
| Cholesterol | 0.005 |
| Ibuprofen | 0.005 |
| Inverse distance to the nearest major road | 0.005 |
| Overall health rating | 0.005 |
| Water intake | 0.005 |
| MET minutes per week for moderate activity | 0.005 |
| Sodium in urine | 0.005 |
| Dried fruit intake | 0.005 |
| Testosterone | 0.005 |
| Alkaline phosphatase | 0.004 |
| Particulate matter air pollution (pm2.5); 2010 | 0.004 |
| Hip pain | 0.004 |
| Summed MET minutes per week for all activity | 0.004 |
| Exposure to tobacco smoke outside home | 0.004 |
| Time spend outdoors in summer | 0.004 |
| Basophill count | 0.004 |
| High light scatter reticulocyte count | 0.004 |
| Family history of heart disease | 0.004 |
| Number of treatments/medications taken | 0.004 |
| Platelet crit | 0.004 |
| Chest pain or discomfort | 0.004 |
| Body fat percentage | 0.004 |
| Traffic intensity on the nearest road | 0.004 |
| Immature reticulocyte fraction | 0.004 |
| Omeprazole | 0.004 |
| Aspartate aminotransferase | 0.003 |
| Total bilirubin | 0.003 |
| Cystatin C | 0.003 |
| Family history of Chronic bronchitis/emphysema | 0.003 |
| Albumin | 0.003 |
| Family history of Bowel cancer | 0.003 |
| HDL cholesterol | 0.003 |
| Reticulocyte percentage | 0.003 |
| Dentures | 0.003 |
| Time spent outdoors in winter | 0.003 |
| Monocyte percentage | 0.003 |
| Basophill percentage | 0.003 |
| Stroke | 0.003 |
| Sum of road length of major roads within 100m | 0.002 |
| Family history of Breast cancer | 0.002 |
| Alcohol drinker status | 0.002 |
| Folic acid or Folate use | 0.002 |
| Cereal intake | 0.002 |
| Inverse distance to the nearest road | 0.002 |
| Back pain | 0.002 |
| Aspirin | 0.002 |
| Sleep duration | 0.002 |
| Platelet count | 0.002 |
| Getting up in morning | 0.002 |
| Fresh fruit intake | 0.002 |
| Laxatives | 0.002 |
| Red blood cell (erythrocyte) distribution width | 0.002 |
| Coffee intake | 0.002 |
| Glucosamine | 0.002 |
| Waist circumference | 0.002 |
| Maternal smoking around birth | 0.002 |
| Loose teeth | 0.002 |
| Lamb/mutton intake | 0.002 |
| Pulse rate | 0.001 |
| Biological Sex | 0.001 |
| Ever had same-sex intercourse | 0.001 |
| Tense / 'highly strung' | 0.001 |
| Alcohol intake frequency. | 0.001 |
| Guilty feelings | 0.001 |
| Miserableness | 0.001 |
| Nucleated red blood cell percentage | 0.001 |
| Headache | 0.001 |
| Sleeplessness / insomnia | 0.001 |
| Frequency of tenseness / restlessness in last 2 weeks | 0.001 |
| Total traffic load on major roads | 0.001 |
| Average total household income before tax | 0.001 |
| Bleeding gums | 0.001 |
| Salad / raw vegetable intake | 0.001 |
| Wears glasses or contact lenses | 0.001 |
| Stomach or abdominal pain | 0.001 |
| Fish oil | 0.001 |
| Non-oily fish intake | 0.001 |
| Heart attack | 0.001 |
| Vitamin C use | 0.001 |
| Monocyte count | 0.001 |
| Neck or shoulder pain | 0.001 |
| Worry too long after embarrassment | 0.001 |
| Asthma | 0.001 |
| Calcium estimated in diet | 0.001 |
| Loneliness, isolation | 0.001 |
| Poultry intake | 0.001 |
| Frequency of unenthusiasm / disinterest in last 2 weeks | 0.001 |
| Morning/evening person (chronotype) | 0.001 |
| Fed-up feelings | 0.001 |
| Paracetamol | 0.001 |
| Wheeze or whistling in the chest in last year | 0.001 |
| Seen general practitioner for nerves, anxiety, tension or depression | 0.001 |
| Falls in the last year | 0.001 |
| Zinc | 0.001 |
| Time spent using computer | 0.001 |
| Angina | 0.001 |
| Selenium | 0.001 |
| Pace-maker | 0.001 |
| Vitamin D use | 0.001 |
| Frequency of solarium/sunlamp use | 0.001 |
| High blood pressure | 0.001 |
| Family history of Lung cancer | 0.001 |
| Family history of Parkinson's disease | 0.001 |
| Cooked vegetable intake | 0.001 |
| Fractured/broken bones in last 5 years | 0.001 |
| Vitamin B use | 0.001 |
| Vitamin A use | 0.001 |
| Worrier / anxious feelings | 0.001 |
| Risk taking | 0.001 |
| Vitamin E use | 0.001 |
| Number of vehicles in household | 0.001 |
| Facial pain | 0.001 |
| Close to major road | 0.001 |
| Number of self-reported cancers | 0.001 |
| Pain all over the body | 0.001 |
| Iron | 0.001 |
| Length of mobile phone use | 0.001 |
| Hearing difficulty/problems with background noise | 0.001 |
| Painful gums | 0.001 |
| Blood clot in the lung | <0.001 |
| Irritability | <0.001 |
| Other eye problems | <0.001 |
| Seen a psychiatrist for nerves, anxiety, tension or depression | <0.001 |
| Education | <0.001 |
| Diabetes diagnosed by doctor | <0.001 |
| Sensitivity / hurt feelings | <0.001 |
| Mood swings | <0.001 |
| Beef intake | <0.001 |
| Comparative body size at age 10 | <0.001 |
| Cancer diagnosed by doctor | <0.001 |
| Daytime dozing / sleeping (narcolepsy) | <0.001 |
| Cheese intake | <0.001 |
| Facial ageing | <0.001 |
| Ever smoked | <0.001 |
| IPAQ activity group | <0.001 |
| Variation in diet | <0.001 |
| Able to confide | <0.001 |
| Long-standing illness, disability or infirmity | <0.001 |
| Skin colour | <0.001 |
| Handedness (chirality/laterality) | <0.001 |
| Snoring | <0.001 |
| Breastfed as a baby | <0.001 |
| Pork intake | <0.001 |
| Weight change compared with 1 year ago | <0.001 |
| Nervous feelings | <0.001 |
| Frequency of depressed mood in last 2 weeks | <0.001 |
| Suffer from 'nerves' | <0.001 |
| Part of a multiple birth | <0.001 |

SHBG = Sex hormone binding globulin, IGF-1 = Insulin-like growth factor 1, MET = Metabolic Equivalent Task, HDL = high-density lipoproteins, IPAQ = International Physical Activity Questionnaire.

**Supplementary Table 2**. A brief description of each of the 20 potential aSAH risk factors studied with the traditional statistical models.

| **Variable name** | **Description** | **Unit of measurement** |
| --- | --- | --- |
| Peak expiratory flow | Peak expiratory flow during blow. | litres/min |
| SHBG | Protein produced by the liver that binds to sex hormones, particularly testosterone and oestrogen, regulating their bioavailability in the bloodstream. | nmol/L |
| C-reactive protein | Substance produced by the liver in response to inflammation, and its levels in the blood increase during inflammatory conditions. | mg/L |
| IGF-1 | A hormone similar in structure to insulin that plays a key role in growth and development by promoting cell division and growth in various tissues. | nmol/L |
| Mean sphered cell volume | Average volume of spherical red blood cells. | femtolitres |
| Sitting height | Distance from rump to crown when sitting, calculated as the difference between seated height and seating box height. | cm |
| Hand grip strength |  | Kg |
| Forced vital capacity | Forced vital capacity value calculated from blow. | litres |
| Glycated haemoglobin (HbA1c) | Form of haemoglobin that is chemically linked to glucose, serving as a marker for long-term blood sugar levels over the previous two to three months. | mmol/mol |
| Haematocrit percentage | Haematocrit Percentage is the relative volume of packed erythrocytes to whole blood, computed by the formula: (red blood cells x mean corpuscular volume) / 10. | % |
| Urea | Nitrogenous compound formed in the liver as a waste product of protein metabolism, which is then excreted from the body through urine. | mmol/L |
| Number of self-reported non-cancer illnesses | Number of non-cancer illnesses entered by interviewer after verbal interview with participant. |  |
| Systolic blood pressure | Blood pressure, automated reading, systolic. Two measures of blood pressure were taken a few moments apart and averaged. | mmHg |
| Townsend deprivation index at recruitment | Townsend deprivation index calculated immediately prior to participant joining UK Biobank. Based on the preceding national census output areas. Each participant is assigned a score corresponding to the output area in which their postcode is located. |  |
| Body mass index (BMI) | BMI value here is constructed from height and weight measured during the initial Assessment Centre visit. Value is not present if either of these readings were omitted. | Kg/m2 |
| Creatinine (enzymatic) in urine | Quantifies the level of creatinine, a muscle metabolism byproduct, using enzymatic assays to assess kidney function and filtration efficiency. | micromole/L |
| Impedance of leg | Body composition estimation by impedance measurement of averaged left leg and right leg impedance. | ohms |
| Lymphocyte count | Result of "Lymphocytes Number" assay, performed on blood sample, obtained from UK Biobank assessment centre visit. Lymphocyte count is the proportion of ( lymphoctyes / 100 ) x white blood cell count. | 10^9 cells/Litre |
| Tea intake | Touchscreen question "How many cups of tea do you drink each DAY? (Include black and green tea)". | cups/day |
| Neutrophil percentage | Result of "Neutrophils Percentage" assay, performed on blood sample, obtained from UK Biobank assessment centre visit. Neutrophils (Percentage) is calculated as the proportion of neutrophils in the leukocytes. | % |

SHBG = Sex hormone binding globulin, IGF-1 = Insulin-like growth factor 1.

**Supplementary Table 3**. The interaction effects between the potentially novel aneurysmal subarachnoid haemorrhage (aSAH) risk factors and the established aSAH risk factors.

| **Variables** | **Mean sphered cell volume OR (95% CI)** | **Haematocrit percentage (95% CI)** | **Log peak expiratory flow (95% CI)** | **Tea intake (95% CI)** |
| --- | --- | --- | --- | --- |
| Age | 1.00 (0.99 - 1.00) | **1.00** (0.99 - 1.00)** | 0.99 (0.97 - 1.01) | 0.99 (0.99 - 1.00) |
| Female Sex | 1.02 (1.00 - 1.05) | 1.04 (1.00 - 1.09) | 0.88 (0.63 - 1.24) | **1.04* (1.01 - 1.09)** |
| Hypertension | 1.02 (1.00 - 1.05) | 1.00 (0.96 - 1.04) | 1.23 (0.91 - 1.66) | 1.00 (0.96 - 1.04) |
| Alcohol Use: Daily | 0.96 (0.96 - 1.03) | 1.00 (0.95 - 1.07) | 0.95 (0.60 - 1.52) | 0.97 (0.91 - 1.04) |
| Alcohol Use: Never | 1.03 (0.98 - 1.07) | 1.00 (0.93 - 1.07) | 0.80 (0.48 - 1.34) | 1.03 (0.98 - 1.08) |
| Alcohol Use: Often | 0.98 (0.95 - 1.02) | 0.99 (0.94 - 1.04) | 0.99 (0.68 - 1.45) | 1.02 (0.97 - 1.07) |
| Alcohol Use: Unknown | 1.13 (0.86 - 1.49) | 0.93 (0.67 - 1.31) | 1.06 (0.09 - 12.36) | 0.82 (0.40 - 1.70) |
| Smoking Status: Current | 1.02 (0.98 - 1.06) | 1.00 (0.95 - 1.05) | 0.77 (0.53 - 1.11) | 1.05 (1.00 - 1.10) |
| Smoking Status: Previous | 0.99 (0.96 - 1.02) | 1.02 (0.98 - 1.07) | 1.14 (0.80 - 1.63) | 1.02 (0.97 - 1.08) |
| Smoking Status: Unknown | 0.95 (0.82 - 1.11) | 0.96 (0.77 - 1.20) | 1.18 (0.18 - 7.73) | 1.14 (0.96 - 1.35) |

All odds ratios indicate the odds-ratio for the interaction effect between the potentially novel aSAH risk factor and the established risk factor. No main effects are shown. *: Statistically significant at a threshold of p<0.05 (indicated in bold). **: Statistically significant at a threshold of p<0.01 (indicated in bold). OR = Odds-ratio, CI = Confidence interval.
